# Making Heat Visible: Promoting Energy Conservation Behaviors Through Thermal Imaging

**DOI:** 10.1177/0013916514546218

**Published:** 2015-12

**Authors:** Julie Goodhew, Sabine Pahl, Tim Auburn, Steve Goodhew

**Affiliations:** 1 Plymouth University, Devon, UK

**Keywords:** energy conservation behavior, energy visibility, thermal imaging, environmental psychology, behavioral interventions

## Abstract

Householders play a role in energy conservation through the decisions they make about purchases and installations such as insulation, and through their habitual behavior. The present U.K. study investigated the effect of thermal imaging technology on energy conservation, by measuring the behavioral effect after householders viewed images of heat escaping from or cold air entering their homes. In Study 1 (*n* = 43), householders who received a thermal image reduced their energy use at a 1-year follow-up, whereas householders who received a carbon footprint audit and a non-intervention control demonstrated no change. In Study 2 (*n* = 87), householders were nearly 5 times more likely to install draught proofing measures after seeing a thermal image. The effect was especially pronounced for actions that addressed an issue visible in the images. Findings indicate that using thermal imaging to make heat loss visible can promote energy conservation.

## Introduction

Making the invisible visible can have a compelling quality, for example, in X rays or night vision images. Modern technologies can convert the invisible into visible formats and it is argued that visualization can have a powerful effect on human behavior (O’Neill, Boykoff, Niemeyer, & Day, 2013). Seeing familiar landscapes under a changed climate promotes consideration of adaptation and mitigation (Sheppard, 2005). Making real-time energy usage visible through portable displays can promote energy conservation (Darby, 2012). Visual communications are not neutral, they provide a more emotive stimulus than text which viewers find themselves forced to engage or disengage with (Joffe, 2008). A viewer’s response to the visual is “subject to alternative interpretations” (Nicholson-Cole, 2005). Images can trigger negative responses, of unease or fear thereby generating defensive psychological reactions, powerlessness, or a desensitized response to the issue (Nicholson-Cole, 2005), so it is important to choose the visualizations carefully. The present research therefore explored the behavioral impact of visualizing heat loss from residential dwellings and the implications for energy conservation. Two studies used the technology of thermal imaging to show householders the heat escaping from their homes (or cold air entering) to investigate any motivational effect on energy saving behavior. The focus of this work is on a novel investigation of the behavioral impact of viewing these images and the potential for such visualizations to promote residential energy conservation measures.

### The context: energy demand reduction and households

Global energy demand remains high despite compelling evidence that greenhouse gas emissions from energy consumption and production are a major contributor to global warming (International Energy Agency, 2011) and that those worldwide energy resources might not be able to meet global energy demand (with implications for energy security). Households account for a significant proportion of that energy consumption; approximately 14% of world delivered energy consumption (U.S. Energy Information Administration, 2010). Among European countries, the United Kingdom is one of the higher consumers of energy per dwelling, and its energy efficiency falls short of the EU average (Department of Energy and Climate Change, 2011). Thirty percent of the United Kingdom’s per capita carbon emissions come from home space heating (Department of Environment, Food and Rural Affairs [DEFRA], 2007b). The United Kingdom provides a useful case study therefore, within which to explore how households react to psychological interventions, as the United Kingdom has “one of the oldest and least efficient housing stocks in Europe” (Boardman et al., 2005, p. 38), and there is indeed opportunity for householders to upgrade their homes. An increase in efficiency measures does not necessarily equate to a reduction in energy demand in households as occupiers change their homes and/or accept improved temperature and comfort levels (Lomas, 2010). However, community groups and local councils are already using thermal imaging technology as a communication medium for encouraging energy conservation (predicated on the assumption that “seeing heat” will indeed promote energy conservation behaviors). Therefore, efficiency measures are used in this study as they are the salient outcomes of a visual intervention. The article concentrates on answering the question, “Does ‘making heat visible’ motivate occupants to adopt retrofit efficiency measures, purchase efficiency equipment upgrades, and employ efficient daily habits?”

### Energy visibility and behavior

The opportunity to capitalize on energy conservation measures exists in the United Kingdom. Although 80% of people in a U.K. survey reported thinking about energy saving behaviors, they report inefficient daily usage habits, with, for example, 52% leaving the heating on when they go out for a few hours (Department of Energy and Climate Change, 2013). In a recent government report, 14 million homes in Britain were not loft insulated up to the recommended maximum level (300 mm) out of a total 27 million homes (Department of Energy and Climate Change, 2012). Given that cost-effective measures are available to improve home energy efficiency, there lies a challenge in raising homeowners’ awareness of inefficiencies and motivating them to act, for example, install draught proofing, adjust heating controls, and close curtains at night.

The “inertia” (Department of Trade and Industry [DTI], 2006) may partly be due to the fact that energy is invisible (Burgess & Nye, 2008; Hargreaves, Nye, & Burgess, 2010). A householder may “know” terms associated with energy use but may have difficulty relating that to specific behaviors (Shove, 2003) and may even be misinformed (Stern, 1992; Wilhite & Ling, 1992). For example, householders tend to overestimate the energy use from visible behaviors (turning on lights) and underestimate less visible uses, for example, energy involved in heating water (Steg, 2008). Knowledge of energy use is predicated on what is experienced: light, heat, convenience (Shove, 1997) rather than the amount of energy required to provide these benefits. Understanding the energy used for space heating may be even trickier. Modern central heating systems use energy in invisible ways (especially when compared with more traditional heating such as open fires where the supply of available fuel can be seen, and users intervene, for example, adding logs for more heat). Modern central heating systems maintain a desired status quo of comfort in homes, responding to pre-determined thermostatic settings rather than direct intervention by the householder. These systems can lead to inefficient use of energy. For example, where a system is programmed by the thermostat to reach a certain temperature in the house, it will continue to heat the house until it achieves this temperature, even if all of the windows and doors are open. It is possible that the occupant could feel warm and comfortable yet be oblivious to the waste of heat or the extra energy demand on the system. Householders cannot directly experience the proportion of energy lost at these times, nor the degree of heat lost generally through walls, through windows without curtains, or fireplaces (Carlsson-Kanyama, Linden, & Eriksson, 2005). This invisibility might affect heat conservation and has implications for communicating heat loss. Yet, householders are amenable to taking actions to conserve heat. In the United States, heating-related retrofit behaviors (weatherizing the home, insulating, and improving glazing) have a high behavioral plasticity (90% plasticity where a well-designed intervention can lead to 90% of homes being weatherized within 10 years; Dietz, Gardner, Gilligan, Stern, & Vandenbergh, 2009). Although care should be taken when applying behavioral findings from North America to the United Kingdom (countries have different energy pricing structures, heating and cooling systems with different fuel mixes), designing interventions which have a high potential to lead to behavior change in combination with a high potential for energy demand reduction seems a sensible aim.

### Tailored, visual interventions

Visual images can be powerful. Visualization methods have previously been employed with householders to illustrate sources of heat loss. The Princeton House Doctor Program used “Smoke sticks” to make draughts visible, with the aim of persuading residents of the value of draught proofing.

Telling people that they are losing a certain percentage of home heat through the cracks around the windows is reasonable, but demonstrating the point by allowing the customer to watch the smoke pour out under doors and over window sills is far more compelling. (Yates & Aronson, 1983, p. 483)

This suggests visualization benefits from people’s natural curiosity in seeing the normally invisible. Which psychological principles underlie successful visualization?

First, having the opportunity to see something which is usually invisible attracts attention (Gardner & Stern, 1996). It is argued that the invisibility of energy makes it an intangible concept (Burgess & Nye, 2008), difficult for people to attend to in the sense that energy use is inconspicuous in everyday activities and secondary to the primary ongoing behaviors. One of the first steps in changing behavior may lie in encouraging people to change their attentional set and actively attend to energy issues (Page & Page, 2011). Images can facilitate cognitive processes of attention and affect and “draw viewers in” (O’Neill et al., 2013, p. 11). Images are assumed to afford vivid representations which can be difficult to communicate via text information (S. E. Taylor & Thompson, 1982). The term “vividness” describes a characteristic of communication. A vivid communication is “likely to attract and hold our attention and to excite the imagination to the extent that it is emotionally interesting, concrete and image provoking, proximate in a sensory, temporal or spatial way” (Nisbett & Ross, 1980, p. 45). Vivid information is presumed to affect people and their judgments by being more available (than competing stimuli) for encoding and therefore for recall, and it has increased imageability and increased emotional involvement (S. E. Taylor & Thompson, 1982). Using vivid communications has been shown to attract attention to energy saving information over less vivid mediums (Gonzales, Aronson, & Costanzo, 1988). Indeed, energy audits, intended to encourage energy conservation among householders, have been shown to vary in their capacity to motivate and capture the full attention of the householder, with vivid, visual and meaningful communications for the householder advocated as more effective (Parnell & Popovic Larsen, 2005). Second, Sheppard (2005) has proposed that visualizations have the quality to make abstract issues concrete and specific. Sheppard’s future scenarios convert abstract ideas (of how a local scene may change under a changing climate) into a set of concrete images (of how that particular street will look, in the town where the viewers live). This approach suggests that visualizations work best when they provide specific rather than general information (Shaw et al., 2009; Sheppard et al., 2009).

Third, specificity is particularly powerful when the information conveyed is *personally* relevant or tailored (Abrahamse, Steg, Vlek, & Rothengatter, 2005, 2007; McKenzie Mohr & Smith, 1999; Nicholson-Cole, 2005). Indeed people find something more noticeable when it has salience for them and the motivation to elaborate can be heightened with personal relevance (Petty & Cacioppo, 1986). Tailored approaches may or may not use visualization. For example, real-time energy display units feed back energy use (mostly electricity), often using numerical forms or visual tools (graphs, lights, fuel tank visual analogies) with some connected to more sophisticated usage analysis programs (Hargreaves et al., 2010). However, this visual information may be augmented when it also has personal relevance, for example, monitoring energy use in one’s own home.

Fourth, it is important not just to communicate the problem, but also to make a link to a solution or range of solutions. Midden and Ham (2009) argued that behaviors are affected more when interventions make a strong link between energy saving action and outcome. Visual communications can act as a metonym conveying cause and effect relationships (O’Neill et al., 2013; Willerton, 2005) such that previously held ideas are considered in new ways (Berger, 1972; Robins, 1996). For example, a real-time portable energy meter is capable of immediately displaying the outcome of turning a specific appliance on. This re-materializing of energy is argued to mediate the relationship between the inconspicuous everyday activity and its energy use, such that “invisible energy becomes connected to a more considered frame of consciousness” (Burgess & Nye, 2008, p. 4458). An action/outcome link can be strengthened by variables such as specificity to the behavior, the person and to the situation (McCalley & Midden, 2002; Midden & Ham, 2009; Petty & Cacioppo, 1986). Interventions based on these principles have been reasonably successful in reducing energy use. Savings in the region of 5% to 15% have been reported following installation of real-time energy display units (Darby, 2006) although Darby (2010) stressed that the devices may not automatically increase energy saving behavior. Tailored energy audits have led to energy savings in the realm of 4% to 12% (Abrahamse et al., 2005). Audits may be tailored and specific to a particular home, but still lack capacity to motivate and capture the attention of the householder, for example, if they are low in vividness or salience (Parnell & Popovic Larsen, 2005).

In sum, previous work suggests that interventions that rely on information and feedback need to address attention, specificity, and personal relevance and provide a direct action/outcome link to maximize the likelihood of success. For home space heating behavior, thermography appears to be a technology that fulfills these criteria.

### Visualizing heat and thermography

**Figure 1.**
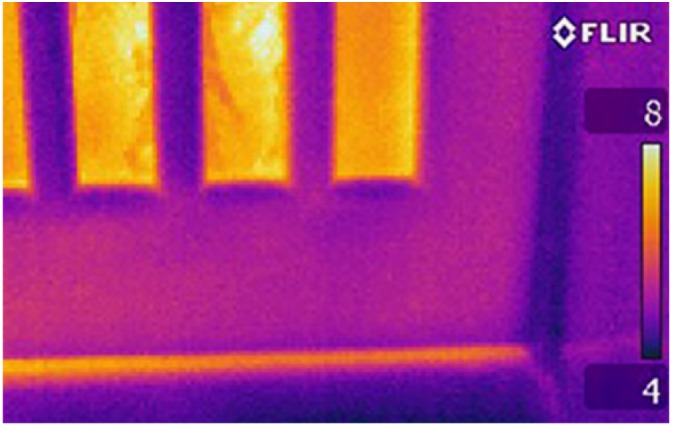
Thermal image showing a draught below the exterior door.

Thermography can be used as a technology to render the normally invisible flow of heat in and around the home visible. Thermal imaging is used primarily to aid in the diagnosis of building defects and can be used as a means of qualitatively inferring heat escape from a building (Pearson, 2011). Thermographic cameras measure infrared radiation from the surface of buildings. Typically, thermal images are taken from the outside of the house on cold sunless evenings (Pearson, 2011; T. Taylor et al., 2012). Different apparent temperatures are then displayed to the user in different colors indicating areas of heat loss and can suggest how energy could be conserved in the home. In Figure 1, the bright area under the closed door indicates a hotter surface temperature than the surrounding area, suggesting that this is where heat is leaking from the house. Installing draught proofing at this point can reduce some of the heat loss from this area, thereby reducing energy use while maintaining thermal comfort. This information is visible and evident to the householder with little deliberation required. Images are unique to each building but nevertheless the kinds of energy conserving actions implicated in thermal images tend to be retrofit behaviors such as loft insulation, wall insulation, draught proofing, improved glazing, improved insulation of windows/doors, and daily use behaviors such as closing windows when the heating is on and not heating unused rooms (Goodhew, Goodhew, Auburn, De Wilde, & Pahl, 2009). These actions offer variable energy savings between 2% and 60% (Energy Savings Trust [EST], 2006).

## Present research

The literature reviewed above suggests that providing engaging, vivid, attention-grabbing tailored energy information can promote voluntary retrofit behaviors and daily use behavior change. If making energy visible does promote energy saving then a thermal image intervention should be better than a non-visually engaging intervention. In two studies, we tested the effect of a thermal imaging intervention compared with a control group. We predicted that householders who saw thermal images of their homes would save energy, as indicated in household bills (Study 1) and take more retrofit actions plus report more daily use energy saving actions (Studies 1 and 2).

## Study 1

Study 1 was designed to test the effect of a thermal image intervention when it was combined with a carbon footprint audit, compared with the behavioral effect from the audit alone and compared with a no intervention control. External thermal images were used for this study.

### Materials and Method

#### Design

The study employed a mixed, between (carbon footprint audit vs. thermal image vs. control) and within-group (Time 1 and Time 2 changes in carbon emissions, energy saving actions) design.

Householders were separated into three groups, two intervention groups and a control group. Measures were obtained once at the start of the study (before the intervention at Time 1) and once after the intervention roughly a year later (Time 2; see Table 1). The first group received an intervention consisting of a carbon footprint audit and thermal image of the home of the householder (thermal image group). The second group was exposed to a carbon footprint audit without thermal images. The third group completed the same measures as both intervention groups but was not exposed to any intervention (control). Table 1 shows the overall design and measures.

**Table 1.** Overview of Design and Measures in Study 1.

	Condition		
Design	Thermal image (*n* = 17)	Carbon footprint (*n* = 17)	Control (*n* = 9)
Time 0	Thermal image taken of home		
Time 1	• New Ecological Paradigm-Revised scale	• New Ecological Paradigm-Revised scale	• New Ecological Paradigm-Revised scale
	• Carbon footprint audit + annual energy usage	• Carbon footprint audit + annual energy usage	• Annual energy usage
	• Energy saving behavior questionnaire	• Energy saving behavior questionnaire	• Energy saving behavior questionnaire
	• Infrared image of home	• Information	
	• Information		
Time 2: ~1 year	• New Ecological Paradigm-Revised scale	• New Ecological Paradigm-Revised scale	• New Ecological Paradigm-Revised scale
	• Carbon footprint audit + annual energy usage	• Carbon footprint audit + annual energy usage	• Annual energy usage

#### Measures

##### Carbon emissions (KgCO_2_) from domestic energy use

Measures of domestic energy use (KgCO_2)_ were taken by using the energy section of a carbon footprint audit, based on the Resurgence carbon calculator (2007). This estimated the household’s carbon footprint from the domestic energy usage data provided in the household’s annual bills. Annual data were taken from two consecutive sets of household fuel bills at Time 1 (before the intervention) and 1 year later (Time 2, after the intervention). All fuels were included. The conversion factors used were 0.43 for electricity and 0.19 for gas (kWh to KgCO_2_, DEFRA, 2007a; The Resurgence Trust, 2007). The carbon footprint also included a section on waste, food miles, and transport but these data were not used in the present study.

##### Energy saving actions adopted

The actions taken by the householders after exposure to the intervention was quantified by counting the overall energy saving retrofit behaviors (a) and daily use behaviors taken by the householder. These behaviors were then divided in to (b) two categories; those actions related to the images or not:

- *Overall energy saving retrofit behaviors and daily use behaviors*. Ten items measured a score for daily energy saving behaviors which the participants were already engaging in (e.g. *I close all curtains at night*). Response scales ranged from *always, frequently, more often than not, occasionally*, to *never*. Higher scores represented more energy saving behaviors. Nine additional questions were asked about the number of retrofit behaviors available to the householder. At Time 2, participants were also asked to describe the behaviors taken since the intervention (Table 4).
- *Separating behaviors directly linked to images versus not linked*. Energy saving behaviors were divided into two types: directly linked to images versus not linked (Table 4). Directly linked behaviors were those where the viewer could have inferred, from the evidence visible in the thermal images, the opportunity to minimize heat loss (improve glazing, insulate the loft, draught proof the door). Not linked behaviors were not visible in the thermal images (installing energy efficiency light bulbs, switching to a renewable heat source, replacing the boiler).

##### Recruitment

Homeowners (N = 43) from a small town in rural England took part who were recruited via advertisements and flyers visible from a stall in a town market, also via advertising in local newspaper articles. Households were allocated to one of the three conditions in a sequential quota fashion: the first householder signed up to the thermal image condition, second householder to the carbon footprint, and third to the control with this pattern then repeating. This was used because those households who responded fast and first (to the adverts and the market stall) could have been more eager to engage with the thermal imaging. Therefore, this sequential method of sign up avoided a “more eager” group being formed and distributed such participants through the three conditions equally. The control group was intentionally smaller to conserve time resources as all of the homes in the study needed to be visited and thermal images taken during the same winter heating season. Therefore, allocation to the control was curtailed once the size of the group reached 11 participants. All participants were offered a thermal image of their home, taken in the winter heating season, to encourage equal “eagerness” between the groups. Only those in the image condition saw their images during the study; the control and carbon footprint householders saw their images upon completion of the study and after all measures had been collected. Again, this method was employed to ensure that all groups were similar in their desire to engage with the thermal images.

Households were kept anonymous for the purpose of the study, in other words, we did not make public which homes had helped in the study or which were being imaged. The householders only saw their own thermal images and we did not share any of these images with other participants or display them locally. In this sense, participants were not aware of who else was involved in the project.

##### Thermal camera and imaging protocol

A FLIR S65 HS infrared camera with wide angle lens captured the thermal images of homes using an iron bar palette to represent the measured surface temperatures. Bright colors represented hotter areas whereas dark colors represented colder areas. Images were taken by a thermographer with a Level 2 certificate in thermography (as defined by the United Kingdom Thermography Association; Snell & Spring, 2008), during the heating season between October and February. To ensure that the images showed just heat loss and not the confounding effects of moisture or solar heating through the day, the images were taken during the winter season, after dark in the evening (one home was imaged very early in the morning before daybreak, but when the heating was on). Householders were instructed to turn their heating on so that a difference of around 10 °C was achieved between the cold outside temperature and the warm inside temperature. Therefore, undesired heat loss or cold ingress would be visible in the images once this differential was achieved. Visits were only taken on days when there was no high sun, precipitation (rain), or high winds (Pearson, 2011). Images were taken of all accessible external walls of the home. Wherever possible the image covered the entire facade, with close up supplementary images taken.

##### Building, demographic, location, and attitude data

Building, demographic data (number of residents per household, age of house), and New Ecological Paradigm-Revised (Dunlap, Van Liere, Mertig, & Jones, 2000) was recorded at Time 1 in the self-report questionnaire (Table 2). None of the homes used air conditioning. The homes were located in close proximity (within a 3-km radius) and were thus exposed to the same weather conditions through the yearlong study. Gas was the main source of fuel for 70% of the sample, with oil being the main source for 14%, electricity for 7%, and wood/other as the fuel in 9% of homes (Table 2). NEP-R scores were collected and are reported here as a measure of participants pro-environmental attitude prior to the study. These data allowed a check for any existing differences between the conditions.

**Table 2.** Building, Demographic, Attitude, and Self-Report Behavior Data Before the Intervention (T1), by Condition (Table 2 shows Means (and Standard Deviation) Unless Otherwise Reported).

	Condition			
	Thermal image (*n* = 17)	Carbon footprint (*n* = 17)	Control (*n* = 9)	Overall mean
Household size	2.35 (1.00)	2.00 (0.87)	2.56 (1.13)	2.26 (0.98)
Median age range of participants	51-60	51-60	41-50	51-60
New Ecological Paradigm-Revised	4.05 (0.50)	4.17 (0.42)	3.94 (0.39)	4.10 (0.08)
Score for daily behaviors already engaged in	4.17 (0.70)	4.27 (0.40)	3.99 (0.42)	4.21 (0.56)
KgCO_2_ at Time 1	4,857 (3,045)	4,742 (3,070)	4,913 (3,450)	4,825 (3,065)
Age of house (years)	65 (42)	60 (40)	39 (28)	57 (42)
Number of retrofit actions available to the householder	4.06 (2.70)	4.35 (2.42)	5.33 (1.80)	4.44 (2.42)
Main fuel source				
Gas	*n* = 12	*n* = 10	*n* = 8	
Electricity	*n* = 1	*n* = 2		
Oil	*n* =2	*n* = 3	*n* = 1	
Wood/other	*n* = 2	*n* = 2		

*Note. n* values for the New Ecological Paradigm-Revised scores are smaller at *n* = 16, *n* = 16, and *n* = 8 as three householders did not complete the scale.

##### Procedure

All householders received an information sheet and were asked for their informed consent to participate. Households in the thermal image condition were visited pre-study to take the thermal images of their home. This visit was arranged with the householder in advance, but householders were shown the thermal images after they had completed the Time 1 measures.

Householders in all conditions were then visited, once to obtain Time 1 measures and show the thermal images where applicable, and once at follow-up a year later (Time 2), see Table 1 for an overview of design and measures. The thermal image was offered free of charge. This may have incentivized people to take part in the study; therefore, all householders were offered this. Participants in the carbon footprint and control groups received their thermal image after all data were collected and the study had ended, that is, after Time 2. The householders in the thermal image condition saw images of their homes on a laptop computer. These were discussed with participants able to make inferences about energy saving from the images, but behaviors were not prompted by the researcher. Upon completion of the data collection, the purpose of the study was explained, remaining questions answered, and a debrief was provided.

### Sample characteristics prior to the intervention

#### Participants

Out of initially 51 householders, we obtained complete data from 43. Of the 8 participants who did not complete the study, 3 did not complete the final questionnaires, 4 could not access their energy usage at Time 2 (T2), and 1 participant moved house (3 non-completers were from the thermal image and carbon footprint group, respectively, and 2 from the control group). Homeowner circumstances did not change in any of the other households.

The sample scored relatively highly on the NEP-R scale and was already engaging in daily energy saving behaviors (Table 2). Energy usage (in KgCO_2_) for the year prior to the intervention is also shown in Table 2. Usage was higher, in all conditions, than the U.K. household average of 4,530 KgCO_2_ (DEFRA, 2008). There were no significant differences between conditions in terms of age of participants, mean number of people in the household, NEP-R attitudes, curtailment behaviors, and intentions to engage in efficiency measures at Time 1 (Table 2).

#### Houses

All houses were detached and had 8 rooms on average (*SD* = 2.91). There were no significant differences between the conditions before the intervention, in the mean age of the homes, nor in mean KgCO_2_ emissions from domestic energy usage. It was noted that the homes in the control condition appeared to be built more recently (although this difference was not statistically significant, *p* = .258). Therefore, an additional analysis, below, compared the opportunities available to the householder to take energy efficiency measures (Table 2).

#### Opportunity for energy efficiency behaviors

The retrofit behaviors that were available to households were counted omitting behaviors already in place or not applicable. For example, some houses were not suitable for cavity wall installation and some houses already had the maximum loft insulation in place. A one-way ANOVA found that households were similar in the number of retrofit behaviors available to them—thermal image, *M* = 4.06; carbon footprint group, *M* = 4.35; control, *M* = 5.33; *F*(2, 42) = 0.83, *p* = .445. This analysis showed that despite somewhat more recent homes, the control group had the same number of opportunities for improving the home.

### Results

#### Carbon emissions from domestic energy usage

**Figure 2.**
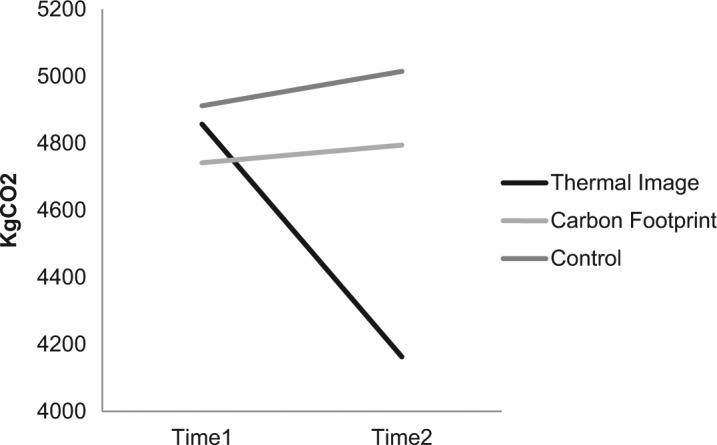
Change in mean annual carbon emission from domestic energy usage, T1 to T2. *Note*. One outlier in energy use was found (in the carbon footprint group) with much higher emissions than other homes (13,963 and 20,997 KgCO_2_). These data were removed from the data set. However, removing this outlier did not affect the overall significance levels reported below nor the conclusions.

Householders in the thermal image group reduced their carbon emissions by 14.29% over the year, a collective saving of 11,799 KgCO_2_, an average reduction of 729.50 KgCO_2_ per household. For the thermal image group, carbon emissions from energy in the home were significantly reduced in the year following the intervention (*M* = 4,163 KgCO_2_), compared with the year previous (*M* = 4,857 KgCO_2_). This was confirmed by a paired-samples *t* test, *t*(16) = 1.79, *p* < .05, one-tailed, with a medium effect size, *r* = .40. The changes for the carbon footprint and control group were not significant—carbon footprint, *t*(15) = −0.17, *p* = .869, +1.12% KgCO_2_, and control group, *t*(8) = −0.44, *p* = .67, +2.09% KgCO_2_ (see Figure 2). Table 3 shows the mean change in carbon emissions per household.^1^

**Table 3.** Carbon Emissions (KgCO_2_) From Domestic Energy Usage.

	Condition		
	Thermal image (*n* = 17)	Carbon footprint (*n* = 16)	Control (*n* = 9)
T1 (year before intervention)			
Total KgCO_2_ per condition	82,577	75,874	44,214
Mean KgCO_2_ per household (*SD*)	4,857 (3,045)	4,742 (3,070)	4,913 (3,450)
T2 (year after intervention)			
Total KgCO_2_ per condition	70,779	76,724	45,139
Mean KgCO_2_ per household (*SD*)	4,163 (2,823)	4,795 (3,547)	5,015 (3,001)

#### Energy saving actions adopted

##### Overall energy saving retrofit and daily use behaviors

Overall, 71 energy saving actions were reported (Table 4), 42 in the thermal imaging group, 21 in the carbon footprint group, and 8 in the control group. Most actions, on average, were taken by the thermal image group (*M* = 2.47), followed by the carbon footprint group (*M* = 1.24) with the least taken by the control group (*M* = 0.89), one-way ANOVA, *F*(2, 42) = 3.56, *p* = .038. Post hoc (Fisher’s Least Significant Difference) tests found a significant difference between the thermal image and carbon footprint group (*p* = .036), the thermal image and control group (*p* = .026), but not between the carbon footprint and control group (*p* = .615).

**Table 4.** Energy Saving Behaviors Taken by Householders, at T2, After the Intervention.

	Number of energy saving behaviors per household		
Type of energy saving behavior	Thermal image (*n* = 17)	Carbon footprint (*n* = 17)	Control (*n* = 9)
Behaviors directly linked to the thermal images			
Installed cavity wall	1	1	0
Installed loft insulation	3	1	2
Under floor insulation	1	0	0
Improved glazing	3	0	1
Installed heavier curtains	0	2	0
Erected porch	2	0	0
Draught proofed windows and doors	1	3	1
Sealed fireplace	5	0	0
Installed reflective radiator panels	1	1	0
Installed radiator valves	1	1	0
Turned off/down appliances	6	6	1
Closed curtains/windows	3	3	0
Mean (SD) number of directly linked behaviors taken per household	1.59 (1.23)	0.53 (0.87)	0.44 (0.73)
Behaviors not directly linked to the thermal images			
Installed boiler	1	0	1
Improved heating system	4	0	0
Maintained heating system	1	0	0
Switch to renewable fuel	4	1	0
Other—Eco car, green tariff	0	0	1
Installed energy efficient light bulbs	2	2	0
Taking shower not bath	1	0	0
Using real-time display unit	2	0	1
Mean number (SD) of non-visible behaviors taken per household	0.59 (0.71)	0.41 (0.87)	0.44 (1.01)
Mean number (SD) of overall energy saving behaviors taken	2.47 (1.66)	1.24 (1.64)	0.89 (1.69)
Total number of energy saving behaviors taken	42	21	8

##### Comparing behaviors directly linked to images versus not linked

Of the behaviors taken (Table 4), some could be, in principle, directly linked to the evidence visible in the thermal images (e.g., the need to improve glazing, insulate the loft, draught proof a door) whereas others could not (e.g., installing energy efficient light bulbs, switching to a renewable source of energy). For example, Figure 1 shows heat leaking underneath a door which can be used by the viewer to infer an opportunity to minimize this heat loss by draught proofing.

**Figure 3.**
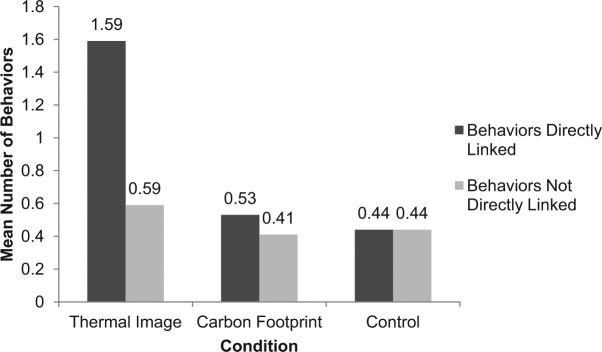
Behaviors directly related to images versus not directly related.

The thermal image group took significantly more energy saving behaviors directly linked to the images (*M* = 1.59) than the carbon footprint group (*M* = 0.53) and control (*M* = 0.44). A two-way mixed ANOVA with condition as the between subjects variable and visibility as the within participants variable showed a marginally significant main effect of condition, *F*(2, 40) = 3.10, *p* = .056, and a significant main effect of visibility, *F*(1, 40) = 6.47, *p* = .015. More importantly, there was a significant interaction between condition and visibility, *F*(2, 40) = 5.24, *p* = .010, $\eta_{\text{p}}^{2}$ = .21. Participants in the thermal image group engaged specifically in those behaviors that were directly linked to the images compared with those not directly linked. No such difference existed in the control and carbon footprint groups (Figure 3).

### Summary study 1

Study 1 showed that the group who saw thermal images of their home made more carbon savings and increased the number of energy saving behaviors they took in the year after the intervention. In comparison, a control group and an audit group made no carbon savings and performed fewer energy saving actions. Specifically, householders who saw the images took more of those actions that were directly linked to evidence visible in the thermal images. These findings support psychological literature that emphasize the need for vivid, visual, tailored interventions (Darby, 2006; Gardner & Stern, 1996; McKenzie Mohr & Smith, 1999; Parnell & Popovic Larsen, 2005).

## Introduction to Study 2

Study 2 was designed to test the effect of a thermal image intervention again, using a methodology that addressed potential limitations of Study 1. First, a larger sample of more geographically spread participants was used. Second, the personal contact of Study 1 was reduced in Study 2, with thermal imaging reports sent to the householders instead of being presented personally by the researcher. In Study 2, all participants received a sum of £500 that they could spend on improving their waste, water, and energy efficiency. Thermal images were taken of the inside of the house as well as the outside, for Study 2. Internal images were arguably more personal. They showed cold air ingress around the householder’s home, were easily recognizable as the house interior, and more specific, as they could be used to infer behaviors related to specific parts of the home (a draught at that door). In addition, the thermal image intervention was compared with an energy audit rather than a carbon footprint audit because Study 1 had shown that thermal images made visible the type of actions normally promoted by an energy audit. An energy audit therefore provided a closer comparison intervention. Again, more energy saving behaviors were expected in the group who saw thermal images compared with the group who did not see thermal images of their home, particularly for those types of issues that are common and can be linked clearly to the images.

## Study 2

Study 2 used an intervention that paired the thermal image with a home energy audit (thermal image group) compared with the home energy audit only intervention (control). This study was a substudy within a wider study (the 21st Century Living Project) in collaboration with the Eden Project, Homebase, and the University of Surrey. All householders taking part in the overall project received a range of sustainability interventions over a year.

### Materials and method

#### Design

The study employed a between (thermal image vs. control) group design. Householders were separated into two groups, one intervention group and a control group. Measures of energy saving actions already in the home were obtained once at the start of the study, before the intervention and new actions captured once after the intervention roughly a year later (follow-up). The first group received an intervention consisting of an energy audit and thermal image of the home of the householder (thermal image group). The second group was exposed to an energy audit without thermal images (control).

#### Measures

Reports were collected from householders by the auditors at their return visit at the end of the 21st Century Living Project. Participants were asked to record all actions taken through the year, along with providing the receipts for connected purchases made during the project. In addition to this, a survey captured these behaviors, including items such as “Have you increased the depth of loft insulation?” “How much of the property is double glazed?” “Are reflective radiator panels present?” By comparing responses at the end of the survey against the response at the outset of the project, it was possible to count energy saving actions taken only after the presentation of the thermal images.

#### Sampling and recruitment

Sample selection of the original 100 participants was made via a national home improvement store’s database of customers who held store loyalty cards. First, any homes that had ever purchased pro-environmental products were deselected from the database to recruit participants who had not previously bought products related to sustainable living. From the remaining cohort, a geographical area was chosen covering England only. Homes were sent an invitation but no mention was made of sustainable living. Participants were offered an incentive of £500 to participate, which could be spent on waste, water, or energy efficiency. Two hundred twenty-six householders initially replied to this offer. Geodemographic profiling was used by the 21st Century Project to select 100 homes from the 226 by matching the sample with the population of U.K. householders and with the proportions of age and type of house in the United Kingdom (Department for Communities and Local Government, 2007). Two final selection criteria were that the participants had to own their own home and have email access. All who volunteered were sent, as a thank you, a voucher for a family visit to the Eden Project in Cornwall. One hundred householders made up the final sample, of which 61 received a thermal image report and 39 did not (providing a control group). Thermal images were taken of homes in the Cornwall, Milton Keynes, Oxford, Sheffield, Devon, Derbyshire, and Birmingham areas of the United Kingdom, whereas homes in the North Devon and Leeds areas served as a control (no images were taken here). Participants were aware that there were 100 homes involved in the project across England, but were not aware which homes had been imaged.

#### Participants

Data on age of participants, socioeconomic background, and type of home are provided in Table 5. All participants were homeowners.

**Table 5.** Study 2: Sample Characteristics, by Condition.

	Thermal image (*n* = 54)	Control (*n* = 33)	Sample mean
Mean number (SD) of residents per household	2.59 (0.14)	2.16 (0.18)	2.44 (0.11)
Mean (SD) age of participants	41 (11.89), *n* = 44	43.35 (10.23), *n* = 26	42.81 (11.90)
New Ecological Paradigm-Revised (*SD*)	3.82 (0.39), *n* = 52	3.88 (0.43), *n* = 32	3.84 (0.41)
Mean (SD) age of home^a^	61 years (41)	68 years (53)	67 (*58*)
Socioeconomic background (*SD*)	C1 (3.26)	C1/C2 (3.64)	C1 (3.36)^1^
	Mode = C1	Mode = C2	Mode = C1
Detached	31.5%	27.3%	30%
Semi	29.6%	36.3%	32%
Mid-terrace	25.9%	21.2%	24%
End terrace	13%	15.2%	14%
Mean number (SD) of actions available to the householder	3.81 (0.67)	3.64 (0.60)	3.75 (0.61)

*Note*. Not all householders completed all of the items on the questionnaires; hence, *n*s vary slightly.

a A cottage of 400 years old was removed from the *t* test analysis for age of home.

1 C1 denotes the social grade based on occupation (range from A to E), using the British National Readership Survey (IPSOS-MORI, 2009)

#### Thermal imaging protocol

**Figure 4.**
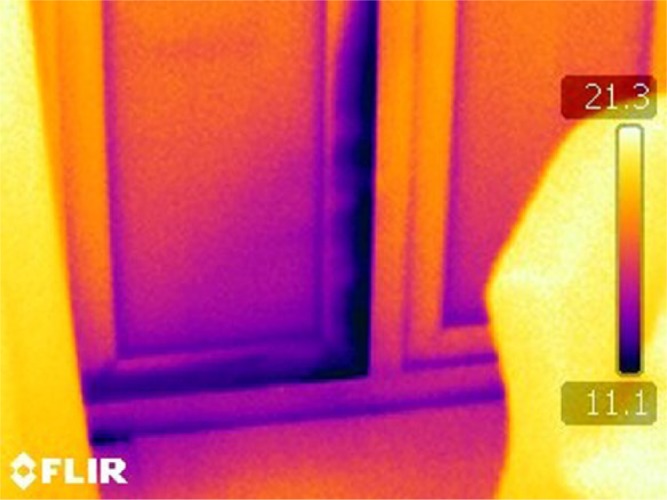
Example of thermal image showing dark (cold) areas where there is cold air ingress around the surround of the doorway which leads outside.

The thermal imaging protocol and equipment was similar to Study 1). However, for this study, images were taken of the internal areas of the home as well as the external facade. Therefore, on an internal image, a dark area will represent cold within the home (see Figure 4). At least two external aspects of the homes were imaged.

#### Procedure

At the outset of the project, all homes were visited by a 21st Century auditor who assessed the home with the householder. Householders were given a free energy monitor, a shopping “bag for life” and shower timer. £500 was given with the instruction that it will be spent on improving the household’s energy, water, or waste efficiency. Between September and the following September, all homes received information on additional aspects of sustainable living such as growing food and water efficiency. This was disseminated via an information pack at the time of the energy audit, via emails and through a project website (this became interactive in February). All householders were told that they may receive a thermal image of their home as part of the project on a day to be arranged during the winter heating season after sunset.

**Figure 5.**
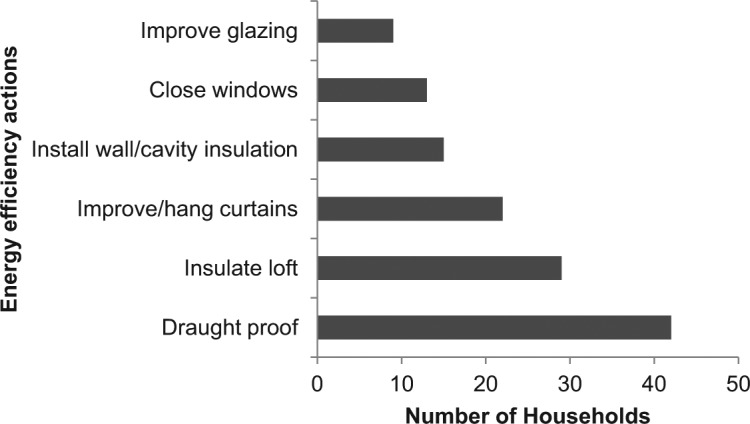
Tailored energy efficiency suggestions evident in the images and advised in the thermographic report.

In January, the thermal imaging of homes began. Householders were contacted and given 7 days’ notice that the thermographer would be visiting. The thermographer also completed an internal “walk about” of the home, imaging areas inside the home where cold air was entering the building. Color images were sent to the householder in the form of an emailed report, and a printed color version was posted. The report started with sample images with general advice on how to interpret the images, then presented the householders’ own images and a short written report containing advice, specific to the images, on how to improve the thermal efficiency of the home (see Figure 5 for an overview of actions recommended; a sample report is available from the first author).

### Sample characteristics

There were no significant between condition differences in NEP-R, type of home, age of participants, socioeconomic background, or home characteristics (see Table 5).

#### Opportunity to take energy efficiency behaviors

Before the intervention, the number of energy saving measures possible in each home was counted. This count omitted those energy efficiency measures which were already in place in the home or not applicable to a particular home. Householders were similar in the number of measures that were available to them, thermal image, *M* = 3.81, control, *M* = 3.64 actions, independent, *t*(85) = 1.32, *p* = .190.

### Results

#### Overall energy saving behaviors

Householders took a total of 87 energy saving behaviors, 62 by the thermal image group and 25 by the control group (Table 6). On average, more behaviors were taken by the thermal image group (*M* = 1.07), than the control group (*M* = 0.73), independent, *t*(85) = 1.70, *p* = .046, one-tailed.

**Table 6.** Energy Saving Behaviors Taken by Condition in Study 2.

	Number of householders	
Type of behavior	Thermal image (*n* = 54)	Control (*n* = 33)
Draught proofed	16	2
Improved curtains, door, porch	4	2
Improved glazing	6	4
Insulated cavity wall	8	3
Insulated loft	28	14
*Total number of behaviors take*	*62*	*25*
Mean number of energy saving behaviors taken per home	1.07	0.73

#### Specific behaviors: Likelihood of draught proofing after seeing the images

The energy saving behavior most frequently recommended in the thermal image reports was draught proofing (Figure 5). Hence, we undertook a separate analysis on draught proofing only. Sixteen householders saw the images and draught proofed their homes, compared with two householders who draught proofed their homes but had not seen the images (Table 6). As these were categorical variables, a logistic regression was used to assess the effect of the image. Using “did the householder see the thermal image” as predictor significantly added to the model in a logistic regression, against a constant only model (χ^2^ = 7.99, *p* = .005, *df* = 1). Nagelkerke’s *R*^2^ of .14 indicated a small relationship between seeing the images and draught proofing the house, with this model predicting 79% of householders. According to the Wald criterion, seeing the thermal image significantly contributed to the householder draught proofing their home (*p* = .02), with the odds ratio of 6.53, which equates to a probability ratio (relative risk) of 4.85 (Osborne, 2006). In other words, those who saw the thermal image were 4.85^2^ times more likely to install draught proofing than those who did not see the thermal images.

### Summary Study 2

Study 2 tested the effect of combining a thermal image with an energy audit against an energy audit only (in the context of a much larger sustainability campaign) and focused on householders who had not previously made sustainability-related purchases. Householders in the thermal image group took more retrofit energy saving behaviors and, in particular, they were nearly 5 times more likely to have draught proofed than those who did not see any images. Notably, this specific effect was only found in the thermal image group even though all householders participated in a yearlong sustainability campaign.

## General Discussion

### Using Psychological Principles to Target Energy Saving in Homes

The present study is novel and innovative because it investigates the behavioral effects that thermal images of homes have, using people’s natural curiosity to see the invisible made visible in an engaging way. In Study 1, householders made more carbon savings and took more energy saving behaviors after seeing a thermal image with a carbon footprint audit of their home. In Study 2, householders were nearly 5 times more likely to take simple draught proofing measures after seeing thermal images of their homes combined with an energy audit. In both studies, comparison control groups received identical treatment but without the thermal imaging element. The studies suggest that householders can be motivated to take energy saving behaviors by directly seeing heat escape or cold air enter their homes.

Our findings suggest that something about the visual communication is causing the householders to behave differently. The conceptual analysis in our introduction summarized several key characteristics that should make an intervention successful, namely, attention-grabbing characteristics including vividness and salience, specificity, personal relevance, and a direct action/outcome link. The thermal images draw attention, they are specific to the home and personally relevant to the householder, and frequently (though not always) they provide a direct visual cue to help viewers infer how energy can be saved in the home. Visual communications are proposed to have the capacity to convey known concepts in new ways (Berger, 1972; O’Neill et al., 2013; Robins, 1996; Sheppard, 2005; Willerton, 2005) and rematerialize energy, enabling the connection with everyday activities to be reconsidered (Burgess & Nye, 2008). Indeed, further analysis in Study 1 showed that behaviors were taken specifically for problems that were directly linked to evidence visible in the thermal images. Simple energy saving behaviors such as draught proofing have high behavioral plasticity (Dietz et al., 2009). People can be persuaded to take these measures and yet the participants in this study were not led to these behaviors by a tailored intervention alone (the energy audit or carbon footprint). This finding therefore supports calls to use visualizations with interventions to promote retrofit behaviors (Darby, 2008; Sheppard, 2005) and to design energy saving interventions which are specific, tailored, and engaging (Gardner & Stern, 1996; Parnell & Popovic Larsen, 2005).

The total KgCO_2_ savings from the thermal image group in Study 1 were in the region of 14%. This is in line with the range of reductions in energy usage observed after similar interventions that make energy visible, such as real-time display units. However, such interventions have targeted mainly electricity use, and this is the first study to target energy use from space heating in this way.

### Limitations

The sample sizes were small in both studies, commensurate with the first attempt to investigate a novel approach. Furthermore, while the visualization group did reduce carbon emissions between Time 1 and Time 2, large within-group variances in emissions data are noted for Study 1. The problem with large variance in energy data and whether it masks any between-group differences has been documented in other energy conservation literature and provides a challenge to statistical analysis (Abrahamse et al., 2007; Brandon & Lewis, 1999). This underlines the need to conduct future research with a larger sample size or with households who have very similar energy usage levels. Future studies might measure pure kWh (if the target is energy demand reduction) as opposed to KgCO_2_; however, Study 1 focused on comparing KgCO_2_ as this is a direct impact measure toward carbon reduction goals (Gatersleben, Steg, & Vlek, 2002). The image groups received the thermal image combined with the carbon footprint in Study 1 and an energy audit in Study 2. It is thus not entirely clear whether the images alone would have the same effect (although the thermal imaging element was the only feature that distinguished the comparison groups and interventions within each study). This is an area for future research. Another limitation is the possibility of participant self-selection involved in these two studies. Participants agreed to take part in a study about how they lived. This type of interest may both predispose a participant to take part in such a study and also predispose them to attend to the information about their home.

### Future Research

Both studies were designed primarily to examine the impact of visualization of heat on householders’ energy saving behaviors, compared with non-visual approaches. To retain this focus, the images were not packaged or framed to maximize psychological impact using other known techniques. Future research could use the thermal imaging intervention combined with framing techniques or consequence strategies. Moreover, McKenzie Mohr & Smith (1999) has advocated designing interventions to provide a direct path from intervention to final behavior, for example, providing a team of professionals to empty the loft and install insulation close to the point of seeing the image. Future work might combine thermal imaging with such interventions.

There is much scope for future work using the thermal imaging technique to uncover the psychological processes leading from the presentation of a stimulus for energy saving to final energy saving behavior. For example, what is the effect when a householder sees a generic image of unknown houses? The persuasive impact might be lost or weakened if the building being viewed is not one with which the viewer interacts. Finally, is there a way of translating savings, for example, in financial cost or in using the language of comfort, or by up scaling and envisioning how this contributes to fighting climate change?

It is not yet clear what exactly it is about visualization that might trigger behavior, nor how a visualization such as this is processed along a psychological pathway toward behavior. The images could be used to investigate these processes.

There is a need for theoretical tests and these findings at the “proof of concept” stage suggest appropriate theories. For example, future work could explore relationships between the visuals and memory processes, examining participants’ post-study elaboration of the images (Kavanagh, Andrade, & May, 2005; May, Andrade, Kavanagh, & Penfound, 2008) or the quality of any recurring memory of the images. Particularly, there is the opportunity to use the images as a research tool to examine the responses of householders to these vivid images and what this can tell about the “black box” of vividness. Are vivid messages more persuasive or does vividness (especially when tailored) provide a stronger or more compelling message less likely to be discounted (Petty & Cacioppo, 1986)? What is the nature of the affect generated by the images?

Finally, the visualizations used were static and not in real time. There may be the potential to explore more dynamic visualizations that represent heat flows, and visualizations that are closer to real-time feedback (see http://www.eviz.org.uk/).

## Conclusion

The present research employed an interdisciplinary team of psychologists and building physicists (Stern, 2011) who were qualified thermographers. It addressed direct energy demand for heat in households, one of the most important targets for emissions reduction as this is characterized by plasticity of behavior combined with a large potential for emissions reduction almost immediately (Gardner & Stern, 2008; Gatersleben et al., 2002; Stern, 2011). On average, people in the thermal imaging group made a 14.29% reduction in KgCO_2_ after 1 year and took more energy saving actions in Study 1. People in Study 2 were nearly 5 times more likely to draught proof their homes than those not exposed to the thermal images. Actual savings and energy saving behaviors were measured and achieved within 1 year in both studies, in contrast to technological “fixes,” which may experience delays in effect due to lack of user acceptance or technology development. In sum, interventions that use visualization technology to target energy conservation measures have the potential to achieve “here and now” energy savings.

## Notes

1. Due to the large variation in energy use between households (Table 3), we chose to use and report paired *t* tests to test our specific hypothesis that the thermal imaging group would reduce their carbon footprint. A repeated-measures ANOVA with Time 1 to Time 2 energy use as the within-participant factor and condition as the between-participant factor found no main effect of time, *F*(1, 39) = 0.71, *p* = .406; no main effect of condition, *F*(2, 39) = 0.07, *p* = .933, and no significant interaction between time and condition, *F*(2, 39) = 1.68, *p* = .200. The graph in Figure 2 shows that the thermal image group was the only group to reduce their KgCO_2_.

2. The authors would like to thank one of the reviewers for recommending this interpretation.
